# GdCl_3_ Attenuates Schistosomiasis japonicum Egg-Induced Granulomatosis Accompanied by Decreased Macrophage Infiltration in Murine Liver

**DOI:** 10.1371/journal.pone.0132222

**Published:** 2015-08-28

**Authors:** Shengsheng Zheng, Qiang Lu, Yuanhong Xu, Xiaonan Wang, Jilong Shen, Wei Wang

**Affiliations:** 1 Department of Pathobiology, Key Laboratories of Zoonoses of Anhui Province, Anhui Medical University, Hefei, 230032, China; 2 Department of Clinical Medicine, Anhui Medical University, Hefei, 230032, China; 3 Department of Laboratory Diagnostics, First Affiliated Hospital of Anhui Medical University, Hefei, 230032, China; Queensland Institute of Medical Research, AUSTRALIA

## Abstract

Early-stage hepatic granuloma and advanced-stage fibrosis are important characteristics of schistosomiasis. The direct consequences of gadolinium chloride (GdCl_3_) in egg-induced granuloma formation have not been reported, although GdCl_3_ is known to block the macrophages. In present study, mice were infected with 15 *Schistosoma japonicum* (*S*. *japonicum*) cercariae and treated with GdCl_3_ (10 mg/kg body weight) twice weekly from day 21 to day 42 post-infection during the onset of egg-laying towards early granuloma formation. Histochemical staining showed that repeated injection of GdCl_3_ decreased macrophages infiltration in liver of mice infected with *S*. *japonicum*. Macrophage depletion by GdCl_3_ during the initial phase attenuated liver pathological injury characterized by smaller granuloma size and decreased immune inflammation as well as less fibrogenesis. In addition, IL-13Rα2 expression was reduced by GdCl_3_ in liver of mice infected with *S*. *japonicum*. The results suggest that GdCl_3_ depleted macrophages, which attenuated helminth infected immune responses involving with IL-13Rα2 signal. These findings would highlight a therapeutic potential via manipulating IL-13Rα2+ macrophage in schistosomiasis.

## Introduction

Schistosomiasis is one of the most important poverty-related health problems, and more than 200 million people are currently infected worldwide [1,2]. In the tropical and subtropical regions, it ranks second among human parasitic diseases [3,4]. The social health and economic burdens for affected populations are poorly measured, despite the incidence of acute and advanced schistosomiasis is significantly reduced in China [5–7]. The major cause of mortality is caused by liver granuloma and progressive fibrosis, which often lead to portal hypertension. However, the key cellular and molecular factors that triggered pathological cascade in schistosomiasis are not well understood, which prevent the therapeutic development that targets for reversing hepatic granulomatosis [8–10].

Schistosome infection induces an increase in the levels of Th2 cytokines such as interleukin (IL)-4, IL-5, and IL-13, among which IL-13 is the dominant effector cytokine of liver fibrogenesis. Kupffer cells as the first macrophage population of the liver [9,11–12]. Macrophages at the boundary of granuloma during schistosoma infection are indispensable to the generation of the Th2 response [13–17]. IL-13 can signal through the IL-13 receptor (R) α2 and type II IL-4 receptor, which both regulate the development of fibrosis. IL-13 and IL-13 receptor complex were critical regulators of disease progression in schistosomiasis [16,18–19]. Moreover, macrophages can produce a pro-fibrogenic transforming growth factor (TGF)-β1 via IL-13Rα2 that is the 'decoy' IL-13 receptor as a key life sustaining 'off' switch for tissue damaging inflammation [20–22]. Macrophages are able to produce a variety of enzymes, cytokines, and mediators that could initiate and/or maintain the inflammatory and immune responses. In this way, we hypothesize that IL-13Rα2 expressing macrophages contribute to the immunopathological development in schistosomiasis.

It is well known that intravenous injection of GdCl_3_, a rare earth metal salt, is able to not only block the phagocytosis of macrophages in liver and spleen, but also eliminate them [23,24]. GdCl_3_ selectively depleted macrophage and used to be a tool for macrophage function research. In the present study, we determined whether GdCl_3_ administration attenuates hepatic immunopathological injury in *S*. *japonicum* murine model.

## Materials and Methods

### Ethics statement

All animal protocols were approved by the Animal Research Committee of the Anhui Medical University at Hefei, China.

### Animals and mice attacked with *S*. *japonicum*

Female BALB/c mice, 6 weeks old, approximately 25 g, were obtained from the Experimental Animal Center of the University of Science and Technology of China (Hefei, China), and housed with free access to food and water. Cercariae of *S*. *japonicum* were released from the Oncomelania hupensis snails (Wuxi, China). The mice were randomly assigned into four groups (n = 6 in each group). Mice were percutaneously infected through abdomen with 15 cercariae of *S*. *japonicum* with GdCl_3_- or saline-injection as described previously [22,25]. Non-infected animals of the same sex and age with GdCl_3_- or saline-injection were used as controls.

### Treatment of mice with GdCl_3_ in vivo

GdCl_3_ solution at a concentration of 2 mg/mL was prepared. Briefly, 0.056 g of GdCl_3_·6H_2_O (Sigma Aldrich; St. Louis, MO, USA) was weighed, and dissolved in 20 mL of saline. The solution was filtered through a 0.22 μm filter, aliquoted, stored at 4°C, and used within a week. Mice were injected with GdCl_3_ 10 mg/kg body weight or saline every 3 days from day 21 to day 42 post-infection by the tail vein as described previously [26]. Twenty-four hours prior to sacrifice, reinforced injection once was performed, then mice were sacrificed and the liver samples were harvested for the following analysis. The whole experiment was repeated twice.

### Histological examination, immunohistochemistry and verification of macrophage depletion in murine liver tissues

The liver tissues were fixed in 4% paraformaldehyde for overnight, embedded in paraffin, and sectioned (3 μm), which sections were used for hematoxylin and eosin (H&E), Masson trichrome and immunohistochemistry staining following the standard protocols. After H&E staining, the single-egg granulomas were counted, and their sizes were calculated in each section. The following formulae were employed for calculation: size = the maximum transverse diameter × the maximum longitudinal diameter; and the mean size of the egg granulomas = the sum of the size of all egg granulomas/the total number of egg granulomas in each section. Eight to ten images per liver section were photographed under an inverted microscope (Nikon 80I, Japan).

Immunohistochemistry studies was performed on paraffin-embedded tissues sections using primary antibodies against F4/80 (1:400; eBioscience, San Diego, CA,), collagen I, collagen III (both 1:200, Bioworld Technology, USA), monoclonal alpha-smooth muscle actin (α-SMA) (1:400; Dako, Carpinteria, CA), and a horseradish peroxidase-labeled secondary antibody. Immunofluorescent staining in liver sections was performed using the primary antibodies against both IL-13Rα2 (1:200; R&D Systems; Minneapolis, USA) and CD68 (1:100; ED-1; AbD Serotec, Oxford, UK) after the antigen retrieval with citric acid buffer. Secondary antibody of Cy3-conjugated donkey anti-goat IgG (Abcam, Cambridge, UK) and fluorescein isothiocyanate (FITC)-conjugated rabbit anti-mouse IgG (Sigma) were applied at a 1:500 dilution, respectively. Nuclei detection was performed with DAPI (Vector Laboratories), and the staining was visualized under a fluorescence microscope (Nikon 80I; Japan). Goat anti-mouse IgG (1:200; Jackson, USA) were used for each primary antibody.

### Real-time PCR for IL-13Rα2 and collagen I mRNA of murine liver tissues

RNA was extracted from whole liver tissue using the RNA extraction kits (Qiagen) according to the manufacturer’s instructions. Complementary DNA was generated from 1 μg of RNA using the Superscript II kit (Invitrogen). The primers and probe were designed by the Shanghai Shinegene Molecular Biotechnology Co., Ltd. (Shanghai, China), and the primers are as follows: IL-13Rα2 sense, 5′-ATG GCT TTT GTG CAT ATC AGA TGC T-3′; antisense, 5′-CAG GTG TGC TCC ATT TCA TTC TAA T-3′. Collagen I sense, 5′-GCC CGG AAG AAT ACG-3′; antisense, 5′-ACA TCT GGG AAG CAA A-3′. GAPDH sense, 5′-GAG GGG CCA TCC ACA GTC TTC-3′; antisense, 5′-CAT CAC CAT CTT CCA GGA GCG-3′[22]. The cycle threshold (Ct) value of the GAPDH gene served as the housekeeping, and the IL-13Rα2 and collagen І expression was normalized to obtain the ^Δ^Ct value. The ^Δ^Ct values for IL-13Rα2 and collagen І were calculated using the ^Δ^Ct value of the mice in the non-infected mice injected with saline as the reference, and the difference in the expression of the IL-13Rα2 and collagen І genes in the other groups was expressed as 2^-ΔΔCt^ [22,27]. All reactions were performed in triplicate. Levels are expressed relative to matched control samples from the same time points.

### Statistical analysis

Data are presented as mean ± standard deviation of the mean. For the data fitting of the approximate normal distribution, one-way analysis of variance was used to compare the differences between groups, while a *q* test (Newman-Keuls test) was performed to compare the pairwise difference between group means. All tests performed were two-sided, with *P* < 0.05 being considered statistically significant.

## Results

### GdCl_3_ treatment decreases F4/80- or CD68-positive signal expression in egg-induced hepatic granuloma

**Fig 1:**
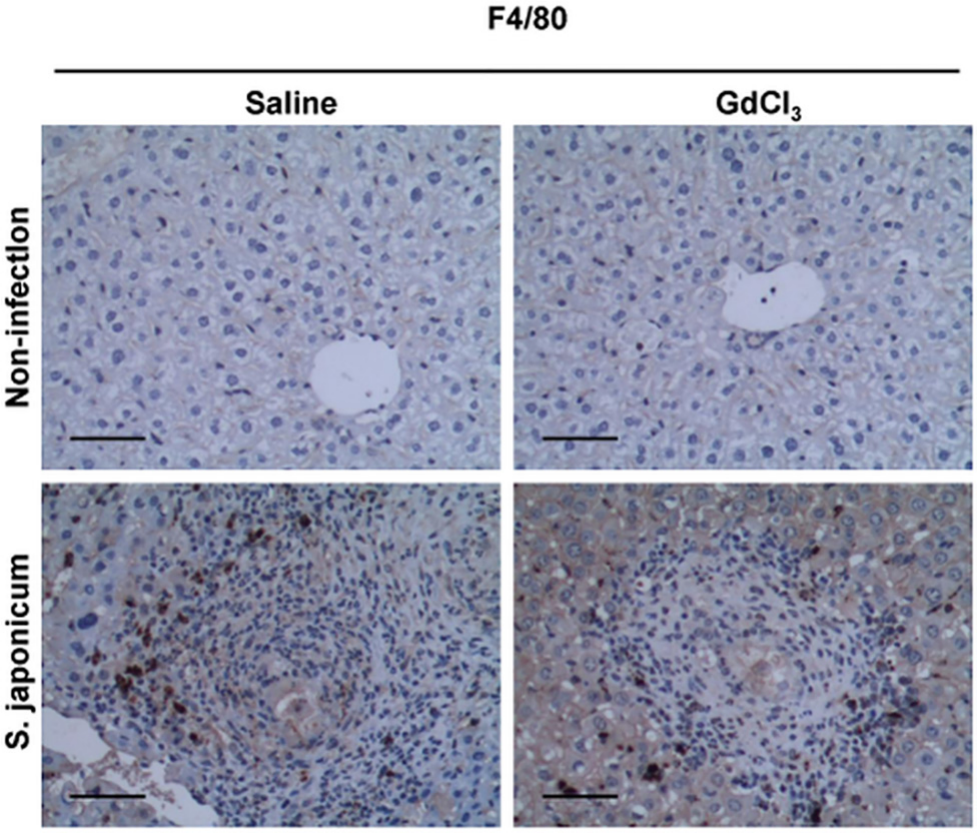
GdCl_3_ injection reduces F4/80 expression in *S*. *japonicum* egg-induced hepatic granuloma. Mice infected with 15 *S*. *japonicum* cercariae and treated with GdCl_3_ (10 mg/kg body weight) or saline twice weekly for 8 times. F4/80 immunohistochemical staining on liver sections prepared from saline-treated mice (control, left column) or GdCl_3_-treated mice (right column) at day 42 post-infection was shown. Original magnification ×200,bar scale 50 μm. Experiments were repeated at least twice with comparable results.

**Fig 2:**
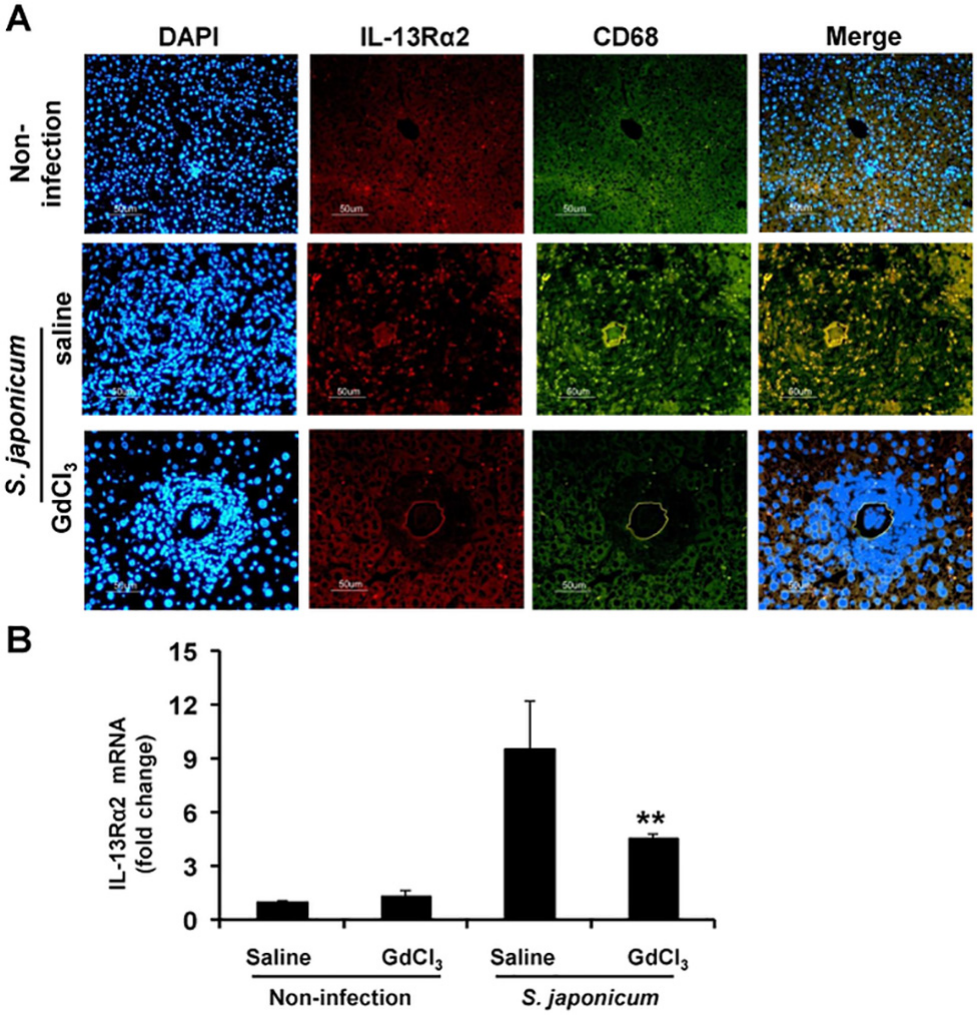
Effect of GdCl_3_ treatment on expression of IL-13Rα2 and CD68 in egg-induced hepatic granuloma. (A) Immunofluorescence double labeled staining was performed to detect IL-13Rα2 (red) and CD68 (macrophages marker, green) in liver sections, with DAPI (blue) conterstain for the nuclei. Merged images were shown in the right panels. Levels of IL-13Rα2 mRNA in livers tissues were measured by real-time PCR (B). ^**^ *P*<0.01, vs infected mice treated with saline. Magnification ×400, bar scale 50 μm.

Anti-mouse F4/80 antibody was used to detect macrophages by the immunohistochemistry staining. As shown in Fig 1, F4/80-positive cells were significantly reduced in GdCl_3_-infected mice, comparing to saline-infected mice. Consistent with the findings that under a fluorescence microscope, many spots highlighted with green fluorescence were observed in saline-infected liver sections (middle row, Fig 2A). Nevertheless, CD68 positive signal (green) were occasionally noticed in GdCl_3_-infected liver sections (bottom row, in Fig 2A). These results indicate that repeated GdCl_3_ injection decreased macrophages infiltration in hepatic granulomatosis.

### GdCl_3_ administration attenuates egg-induced hepatic granuloma inflammation

**Fig 3:**
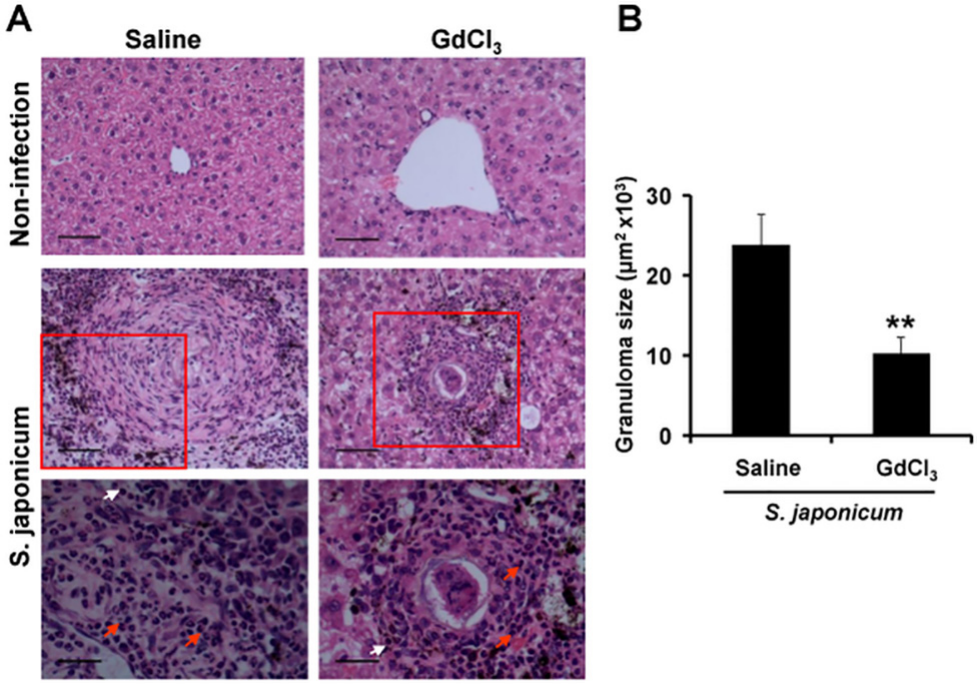
Repeated injection of GdCl_3_ attenuates *S*. *japonicum* egg-induced hepatic granuloma inflammation. Liver sections were stained with H&E (A) for eosinophils (red arrow) and lymphocytes (white arrow) (Original magnification, top and middle row ×200,bar scale 50 μm, bottom row ×400,bar scale 25 μm) or average granuloma sizes (B). Means and SD are shown. ^**^ *P*<0.01.

To investigate the effect of GdCl_3_ on early egg-induced granuloma formation, the mice were sacrificed after 8 times injection of GdCl_3_ by tail vein. Histochemical staining revealed that liver sections were indistinguishable between GdCl_3_- and saline-treated control non-infected mice by H&E staining (Fig 3A). While most of the cells around egg-miracidia granuloma were eosinophils in infected liver sections treated with saline or GdCl_3_ (red arrow, Fig 3A). More importantly, the granuloma size (Fig 3B) in GdCl_3_-infected mice (10.25±2.1 μm^2^× 10^3^) was significantly smaller than that in saline-infected mice (23.87± 3.86 μm^2^× 10^3^) (*P* < 0.05). Therefore, depletion of macrophages of GdCl_3_ attenuates hepatic granuloma inflammation.

### GdCl_3_ injection attenuates hepatic fibrogenesis

**Fig 4:**
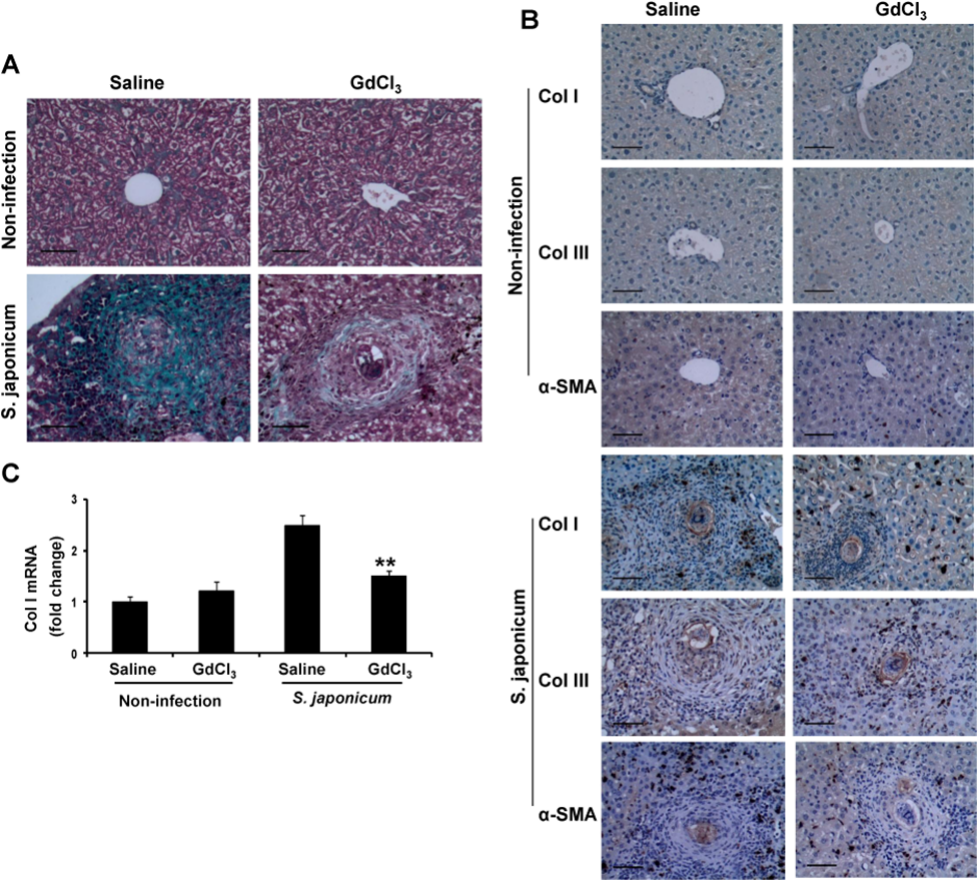
Repeated injection of GdCl_3_ reduced egg-induced hepatic fibrogenesis. Liver sections of saline-treated mice (control, left column) or GdCl_3_-treated mice (right column) were used for Masson trichrome staining for collagen deposition (A). Immunohistochemical staining was performed for collagen I (top row), collagen III (middle row), α-SMA (bottom row) (B). Collagen I mRNA in livers tissue was measured by real-time PCR (C). ^**^ *P*<0.01, vs infected mice treated with saline. Original magnification ×200,bar scale 50 μm.

Hepatic fibrogenesis is one of the features of chronic schistosomiasis [10]. Next to investigate the effect of GdCl_3_ on hepatic fibrosis, collagen deposition were stained with Masson trichrome, and expression of collagen isoforms of collagens I, III and α-SMA were examined by immunohistochemical staining shown in Fig 4. The large, thick, and flame-like fibers surrounding the granulomas and extending outward was observed in saline-infected mice, which was markedly reduced in GdCl_3_-infected mice (Fig 4A). The positive area of collagen I, III, and α-SMA was significantly reduced in GdCl_3_-infected mice, comparing to saline-infected mice, respectively (Fig 4B). Consistently, the level of collagen I mRNA was significantly decreased in GdCl_3_-infected mice compared with control mice (Fig 4C). Hence, GdCl_3_ reduces egg-induced hepatic fibrogenesis in granulomas.

### GdCl_3_ treatment has no effect on worm load, egg burden

Worm pairs, total worms, and total parasite eggs contained similar mature miracidia in the livers of GdCl_3_- and saline-infected mice, which were not significantly different (Table 1). Hence decreased hepatic granulomatous inflammatory and fibrogenesis by GdCl_3_ were not resulted from differences in worm load and egg burden.

**Table 1:** Parasitological measurements in Schistosomiasis japonicum mice treated with GdCl_3_.

Group	No	Total warms	Warms pairs	Total liver eggs (×10^3^)	Size of granulomas (μm^2^×10^3^)
Saline	6	10.0士1.26	5.2士0.49	38.25士4.72	23.87± 3.86
GdCl_3_	6	10.0士2.08	5.0士1.01	38.27士5.89	10.25±2.1

### Reduction of IL-13Rα2 expression in GdCl_3_-infected liver tissues

Our previous study shows enhanced IL-13Rα2 expression in primary macrophages of murine schistosomiasis [22]. Then, we further determined whether depleted macrophages of GdCl_3_ resulted in any differences in IL-13Rα2 expression. Liver sections were detected by double labeled staining of IL-13Rα2 and CD68. In saline-infected mice (middle row, Fig 2A), there was some scattered red coffee bean-like staining, indicating specific IL-13Rα2 positive signals (red). Green coffee bean-like staining was donated to CD68 positive signals (green). Merge in white were demonstrated co-expression of IL-13Rα2 and CD68 at the same position of egg-induced hepatic granuloma (white, in Fig 2A). Surprisingly, the co-expression of the CD68^+^ (green) and IL-13Rα2^+^ (red) signal diminished simultaneously in GdCl_3_-infected mice (bottom row, Fig 2A). Further, the hepatic IL-13Rα2 mRNA expression was analyses by TaqMan PCR. IL-13Rα2 mRNA expression was not significantly changed between GdCl_3_- and saline-treated normal/non-infected mice (*P* > 0.05). However, level of IL-13Rα2 mRNA in saline-infected mice was significantly augmented (9-fold), which was reduced in GdCl_3_-infected mice (5-fold) (*P* < 0.05) (Fig 2B). Overall, depletion of macrophages by injection of GdCl_3_ reduced IL-13Rα2 expression in murine *S*. *japonicum* liver.

## Discussion

Accumulating evidence has shown that macrophages are able to promote, restrict, or resolve inflammation and fibrosis [28–30]. It has been shown that monocytes/macrophages are not only responsible for fibrosis progression, but also for the resolution of hepatic inflammation and fibrosis (for fibrosis regression) [31,32]. For example, the findings using mice genetically defective in macrophage function have confirmed that these cells are essential to normal wound healing, because their depletion results in retarded and abnormal repair [33]. This may due to the function of macrophages to produce anti-inflammatory mediators and matrix metalloproteinases. However, the signaling pathway resulted from changes in gene expression pattern mechanisms of macrophage in the regulation of hepatic pathological development in schistosomiasis is complex [31,32].

GdCl_3_ is nontoxic to other cells and used as a magnetic resonance imaging contrast agent in clinical medicine. GdCl_3_ can induce macrophage inactivation or dormancy as well as macrophage apoptosis [34–36]. To explore the role of IL-13Rα2-expressing macrophages, the current study was in search of GdCl_3_ to selectively deplete macrophages following with effect on IL-13Rα2 expression. Helminth worms live in the portal venous system, and begin to lay eggs after 4 weeks post-infection. In this way, we have designed to administer GdCl_3_ on day 21 post-infection before the initial egg-induced immune responses. Macrophage depletion during the initial phase attenuated liver pathological injury, which is reflected on smaller granuloma size and decreased immune inflammation as well as less fibrogenesis. This is in agreement with the findings that targeting Kupffer cells by GdCl_3_ ameliorates carbon tetrachloride-induced liver fibrosis [37,38], and that pharmacological inhibition of the chemokine CCL2 diminishes liver macrophage infiltration thereby attenuating steatohepatitis during chronic hepatic injury [39]. Thus, macrophage depletion in the initial stage protects against *S*. *japonicum* egg-induced hepatic granuloma formation and collagen deposition. Further study is required to determine the effect of macrophage depletion on the granulomatosis and collagen deposition during the resolution phase.

In chronic stage, schistosomes down-regulate host immune response, which promotes their survival as well as limits the pathological changes in hosts [40,41]. A mixed Th1/Th2 response or slightly biased Th1 response appear to be beneficial by minimizing fibrosis and protecting the host against intestinal and hepatic damage during chronic *S*. *mansoni* infection [19]. IL-13 is a potential therapeutic target for various diseases, such as asthma and ulcerative colitis [42]. IL-13 can also directly induces expression of collagen I and other critical fibrosis-associated genes, e.g, α-SMA and connective tissue growth factor, in hepatic stellate cells [43–45]. IL-13 binds to a receptor complex of IL-4Rα and two IL-13-binding proteins (IL-13Rα1 and IL-13Rα2). These receptors have different affinities to IL-13, participate in different signaling pathways in different contexts [46]. In general, IL-13Rα1 pairs with IL-4α forming a functional receptor for IL-13 that signals and activates the downstream JAK/Stat6 pathway [47]. In contrast, IL-13Rα2 acts as a decoy receptor and has a short cytoplasmic tail that binds IL-13 with 100-fold higher affinity than IL-13Rα1, which inhibits the biological action of IL-13 [48]. Interestingly, the mice with genetic deletion of IL-4Rα in macrophages die due to severe intestinal and liver pathology during acute *S*. *mansoni* infection [49]. Intravenous injection of exogenous soluble IL-13Rα2 protein significantly reduces the volume of granulomas in IL-13Rα2 knockout mice with schistosomiasis [50]. It is interesting to note that IL-13Rα2 gene silencing or IL-13Rα2 signal pathway blockade leads to marked down-regulation of TGF-β1 production and collagen deposition in lung fibrogenesis and allograft fibrosis [21,51]. This is corroborated well with our findings that the expression of IL-13Rα2 was significantly increased in liver macrophages in response to *S*. *japonicum* cercariae infection [22]. The protection against hepatic granulomatosis and collagen deposition by GdCl_3_ is associated with reduced expression of IL-13Rα2 in macrophages. These findings suggest that increased expression of IL-13Rα2 in macrophage plays an important role in triggering hepatic fibrogenesis in response to *S*. *japonicum* cercariae infection. However, it remains unknown if IL-13Rα2 reduction in macrophage decreases TGF-β1 expression. The role of IL-13Rα2 positive macrophages need to be intensively studied. The experiments using mice with macrophage specific knockout of IL-13Rα2 will help to unravel the causal role of macrophage IL-13Rα2 in *S*. *japonicum* egg-induced liver injury, despite global knockout of IL-13Rα2 aggravates granulomatous inflammation and reduces host survival in schistosomiasis [50].

## References

[1] King CH. Parasites and poverty: the case of schistosomiasis. Acta Trop. 2010;113: 95–104. 10.1016/j.actatropica.2009.11.012 19962954 PMC2812649

[2] Tucker MS, Karunaratne LB, Lewis FA, Freitas TC, Liang YS. Schistosomiasis. Curr Protoc Immunol. 2013 11 18;103:Unit 19.1. 10.1002/0471142735.im1901s103 24510597

[3] Greenwald B. Schistosomiasis: implications for world travelers and healthcare providers. Gastroenterol Nurs. 2005;28: 203–205; quiz 206–207. 15976562 10.1097/00001610-200505000-00002

[4] Hu Y, Xiong C, Zhang Z, Luo C, Cohen T, Gao J, et al Changing patterns of spatial clustering of schistosomiasis in Southwest China between 1999–2001 and 2007–2008: assessing progress toward eradication after the World Bank Loan Project. Int J Environ Res Public Health. 2014; 11: 701–712. 10.3390/ijerph110100701 24394217 PMC3924469

[5] Zhou XN, Wang LY, Chen MG, Wu XH, Jiang QW, Chen XY, et al The public health significance and control of schistosomiasis in China—then and now. Acta Trop. 2005; 96: 97–105. 16125655 10.1016/j.actatropica.2005.07.005

[6] Dang H, Xu J, Li SZ, Cao ZG, Huang YX, Wu CG, et al Monitoring the transmission of Schistosoma japonicum in potential risk regions of China, 2008–2012. Int J Environ Res Public Health. 2014;11: 2278–2287. 10.3390/ijerph110202278 24566053 PMC3945598

[7] Chen MG. Assessment of morbidity due to Schistosoma japonicum infection in China. Infect Dis Poverty. 2014;3: 6 10.1186/2049-9957-3-6 24529186 PMC3928580

[8] Magalhaes A, Miranda DG, Miranda RG, Araujo MI, Jesus AA, Silva A, et al Cytokine profile associated with human chronic schistosomiasis mansoni. Mem Inst Oswaldo Cruz. 2004;99: 21–26. 15486630 10.1590/s0074-02762004000900004

[9] Wynn TA, Thompson RW, Cheever AW, Mentink-Kane MM. Immunopathogenesis of schistosomiasis. Immunol Rev. 2004;201: 156–167. 15361239 10.1111/j.0105-2896.2004.00176.x

[10] Andrade ZA. Schistosomiasis and liver fibrosis. Parasite Immunol. 2009;31: 656–663. 10.1111/j.1365-3024.2009.01157.x 19825105

[11] Kolios G, Valatas V, Kouroumalis E. Role of Kupffer cells in the pathogenesis of liver disease. World J Gastroenterol. 2006;12: 7413–7420. 17167827 10.3748/wjg.v12.i46.7413 PMC4087584

[12] Bilzer M, Roggel F, Gerbes AL. Role of Kupffer cells in host defense and liver disease. Liver Int. 2006;26: 1175–1186. 17105582 10.1111/j.1478-3231.2006.01342.x

[13] Schuppan D, Kim YO. Evolving therapies for liver fibrosis. J Clin Invest. 2013; 123: 1887–1901. 10.1172/JCI66028 23635787 PMC3635731

[14] Smith P, Walsh CM, Mangan NE, Fallon RE, Sayers JR, McKenzie AN, et al Schistosoma mansoni worms induce anergy of T cells via selective up-regulation of programmed death ligand 1 on macrophages. J Immunol. 2004;173: 1240–1248. 15240716 10.4049/jimmunol.173.2.1240

[15] Ragheb S, Boros DL. Characterization of granuloma T lymphocyte function from Schistosoma mansoni-infected mice. J Immunol. 1989;142: 3239–3246. 2523427

[16] Liu Y, Munker S, Mullenbach R, Weng HL. IL-13 Signaling in Liver Fibrogenesis. Front Immunol. 2012;3: 116 10.3389/fimmu.2012.00116 22593760 PMC3349963

[17] Kaplan MH, Whitfield JR, Boros DL, Grusby MJ. Th2 cells are required for the Schistosoma mansoni egg-induced granulomatous response. J Immunol. 1998;160: 1850–1856. 9469446

[18] Tacke F, Zimmermann HW. Macrophage heterogeneity in liver injury and fibrosis. J Hepatol. 2014; 60:1090–1096. 10.1016/j.jhep.2013.12.025 24412603

[19] Barron L, Wynn TA. Macrophage activation governs schistosomiasis-induced inflammation and fibrosis. Eur J Immunol. 2011;41: 2509–2514. 10.1002/eji.201141869 21952807 PMC3408543

[20] Friedman SL. Mac the knife? Macrophages- the double-edged sword of hepatic fibrosis. J Clin Invest. 2005;115: 29–32. 15630440 10.1172/JCI23928 PMC539205

[21] Fichtner-Feigl S, Strober W, Kawakami K, Puri RK, Kitani A. IL-13 signaling through the IL-13alpha2 receptor is involved in induction of TGF-beta1 production and fibrosis. Nat Med. 2006;12: 99–106. 16327802 10.1038/nm1332

[22] Wang W, Shen YX, Li J, Zhang SH, Luo QL, Zhong ZR, et al Enhanced expression of the decoy receptor IL-13Ralpha2 in macrophages of Schistosoma japonicum-infected mice. Chin Med J (Engl). 2009;122: 1650–1654. 19719966

[23] Hardonk MJ, Dijkhuis FW, Hulstaert CE, Koudstaal J. Heterogeneity of rat liver and spleen macrophages in gadolinium chloride-induced elimination and repopulation. J Leukoc Biol. 1992;52: 296–302. 1522388 10.1002/jlb.52.3.296

[24] Husztik E, Lazar G, Parducz A. Electron microscopic study of Kupffer-cell phagocytosis blockade induced by gadolinium chloride. Br J Exp Pathol. 1980;61: 624–630. 7459256 PMC2041618

[25] Qi Y, Song XR, Shen JL, Xu YH, Shen Q, Luo QL, et al Tim-2 up-regulation and galectin-9-Tim-3 pathway activation in Th2-biased response in Schistosoma japonicum infection in mice. Immunol Lett. 2012;144: 60–66. 10.1016/j.imlet.2012.03.007 22469568

[26] Van Rooijen N, Sanders A. Kupffer cell depletion by liposome-delivered drugs: comparative activity of intracellular clodronate, propamidine, and ethylenediaminetetraacetic acid. Hepatology. 1996;23: 1239–1243. 8621159 10.1053/jhep.1996.v23.pm0008621159

[27] Livak KJ, Schmittgen TD. Analysis of relative gene expression data using real-time quantitative PCR and the 2(-Delta Delta C(T)) Method. Methods. 2001;25: 402–408. 11846609 10.1006/meth.2001.1262

[28] Mosser DM, Edwards JP. Exploring the full spectrum of macrophage activation. Nat Rev Immunol. 2008;8: 958–969. 10.1038/nri2448 19029990 PMC2724991

[29] Martinez FO, Helming L, Gordon S. Alternative activation of macrophages: an immunologic functional perspective. Annu Rev Immunol. 2009;27: 451–483. 10.1146/annurev.immunol.021908.132532 19105661

[30] Wynn TA, Barron L. Macrophages: master regulators of inflammation and fibrosis. Semin Liver Dis. 2010;30: 245–257. 10.1055/s-0030-1255354 20665377 PMC2924662

[31] Duffield JS, Forbes SJ, Constandinou CM, Clay S, Partolina M, Vuthoori S, et al Selective depletion of macrophages reveals distinct, opposing roles during liver injury and repair. J Clin Invest. 2005;115: 56–65. 15630444 10.1172/JCI22675 PMC539199

[32] Chuah C, Jones MK, Burke ML, McManus DP, Gobert GN. Cellular and chemokine-mediated regulation in schistosome-induced hepatic pathology. Trends Parasitol. 2014;30:141–50. 10.1016/j.pt.2013.12.009 24433721

[33] Lucas T, Waisman A, Ranjan R, Roes J, Krieg T, Müller W, et al Differential roles of macrophages in diverse phases of skin repair. J Immunol. 2010;184: 3964–3977. 10.4049/jimmunol.0903356 20176743

[34] Zeisberger SM, Odermatt B, Marty C, Zehnder-Fjällman AH, Ballmer-Hofer K, Schwendener RA. Clodronate-liposome-mediated depletion of tumour-associated macrophages: a new and highly effective antiangiogenic therapy approach. Br J Cancer. 2006;95: 272–281. 16832418 10.1038/sj.bjc.6603240 PMC2360657

[35] Tyner JW, Uchida O, Kajiwara N, Kim EY, Patel AC, O'Sullivan MP, et al CCL5-CCR5 interaction provides antiapoptotic signals for macrophage survival during viral infection. Nat Med. 2005;11: 1180–1187. 16208318 10.1038/nm1303 PMC6322907

[36] Mizgerd JP, Molina RM, Stearns RC, Brain JD, Warner AE. Gadolinium induces macrophage apoptosis. J Leukoc Biol. 1996;59: 189–195. 8603991

[37] Muriel P, Escobar Y. Kupffer cells are responsible for liver cirrhosis induced by carbon tetrachloride. J Appl Toxicol. 2003;23: 103–108. 12666154 10.1002/jat.892

[38] Rivera CA, Bradford BU, Hunt KJ, Adachi Y, Schrum LW, Koop DR, et al Attenuation of CCl(4)-induced hepatic fibrosis by GdCl(3) treatment or dietary glycine. Am J Physiol Gastrointest Liver Physiol. 2001;281: G200–207. 11408273 10.1152/ajpgi.2001.281.1.G200

[39] Baeck C, Wehr A, Karlmark KR, Heymann F, Vucur M, Gassler N, et al Pharmacological inhibition of the chemokine CCL2 (MCP-1) diminishes liver macrophage infiltration and steatohepatitis in chronic hepatic injury. Gut. 2012;61: 416–426. 10.1136/gutjnl-2011-300304 21813474

[40] Dunne DW, Cooke A. A worm's eye view of the immune system: consequences for evolution of human autoimmune disease. Nat Rev Immunol. 2005;5: 420–426. 15864275 10.1038/nri1601

[41] Anthony RM, Rutitzky LI, Urban JF Jr, Stadecker MJ, Gause WC. Protective immune mechanisms in helminth infection. Nat Rev Immunol. 2007;7: 975–987. 18007680 10.1038/nri2199 PMC2258092

[42] Mentink-Kane MM, Wynn TA. Opposing roles for IL-13 and IL-13 receptor alpha 2 in health and disease. Immunol Rev. 2004;202: 191–202. 15546394 10.1111/j.0105-2896.2004.00210.x

[43] Sugimoto R, Enjoji M, Nakamuta M, Ohta S, Kohjima M, Fukushima M, et al Effect of IL-4 and IL-13 on collagen production in cultured LI90 human hepatic stellate cells. Liver Int. 2005;25: 420–428. 15780068 10.1111/j.1478-3231.2005.01087.x

[44] Weng HL, Liu Y, Chen JL, Huang T, Xu LJ, Godoy P, et al The etiology of liver damage imparts cytokines transforming growth factor beta1 or interleukin-13 as driving forces in fibrogenesis. Hepatology. 2009;50: 230–243. 10.1002/hep.22934 19441105

[45] Liu Y, Meyer C, Müller A, Herweck F, Li Q, Müllenbach R, et al IL-13 induces connective tissue growth factor in rat hepatic stellate cells via TGF-beta-independent Smad signaling. J Immunol. 2011;187: 2814–2823. 10.4049/jimmunol.1003260 21804025

[46] Donaldson DD, Whitters MJ, Fitz LJ, Neben TY, Finnerty H, Henderson SL, et al The murine IL-13 receptor alpha 2: molecular cloning, characterization, and comparison with murine IL-13 receptor alpha 1. J Immunol. 1998;161: 2317–2324. 9725226

[47] Hershey GK. IL-13 receptors and signaling pathways: an evolving web. J Allergy Clin Immunol. 2003;111: 677–690; quiz 691. 12704343 10.1067/mai.2003.1333

[48] Bernard J, Treton D, Vermot-Desroches C, Boden C, Horellou P, Angevin E, et al Expression of interleukin 13 receptor in glioma and renal cell carcinoma: IL13Ralpha2 as a decoy receptor for IL13. Lab Invest. 2001;81: 1223–1231. 11555670 10.1038/labinvest.3780336

[49] Herbert DR, Hölscher C, Mohrs M, Arendse B, Schwegmann A, Radwanska M, et al Alternative macrophage activation is essential for survival during schistosomiasis and downmodulates T helper 1 responses and immunopathology. Immunity. 2004;20: 623–635. 15142530 10.1016/s1074-7613(04)00107-4

[50] Mentink-Kane MM, Cheever AW, Thompson RW, Hari DM, Kabatereine NB, Vennervald BJ, et al IL-13 receptor alpha 2 down-modulates granulomatous inflammation and prolongs host survival in schistosomiasis. Proc Natl Acad Sci U S A. 2004;101: 586–590. 14699044 10.1073/pnas.0305064101 PMC327191

[51] Brunner SM, Schiechl G, Kesselring R, Martin M, Balam S, Schlitt HJ, et al IL-13 signaling via IL-13Ralpha2 triggers TGF-beta1-dependent allograft fibrosis. Transplant Res. 2013;2: 16 10.1186/2047-1440-2-16 24143891 PMC4016099

